# The Effect of Weed Control with Pre-Emergence Herbicides on the Yield Level of Mung Bean Yield

**DOI:** 10.3390/plants14020275

**Published:** 2025-01-18

**Authors:** Cailing Jing, Junying Wang, Yonghong Wu, Yufan Zhou, Huijun Zhu, Yaowen Zhang, Shuqi Dong, Xiaorui Li, Juan Zhao, Junli Cao, Xiangyang Yuan, Xi’e Song

**Affiliations:** 1 College of Agriculture, Shanxi Agricultural University, Jinzhong 030801, China; j10237226@163.com (C.J.); 15225547779@163.com (J.W.); wyh2024920@163.com (Y.W.); zhouyufan0830925@163.com (Y.Z.); zyw8118571@126.com (Y.Z.); dong-s-q@163.com (S.D.); lixiaorui@sxau.edu.cn (X.L.); sxndzhaojuan@163.com (J.Z.); yuanxiangyang200@163.com (X.Y.); 2 Shanxi Center for Testing of Functional Agro-Products, Shanxi Agricultural University, Jinzhong 030031, China; nkyzjs@126.com

**Keywords:** pre-emergence herbicide, green beans, yield, weeds, physiological indicators, soil enzyme activity

## Abstract

The mung bean (*Vigna radiata* (Linn) Wilczek.) is a major grain crop in China, but its yield is significantly impacted by weeds. However, no pre-emergence herbicides are registered for mung bean fields in the China Pesticide Information Network. Screening for efficient and safe pre-emergence herbicides could improve mung bean production efficiency. In this study, six pre-emergence herbicides were selected: 480 g/L alachlor (1935.00 g a.i ha^−1^), 720 g/L metolachlor (1620.00 g a.i ha^−1^), 100 g/L imazethapyr (100.50 g a.i ha^−1^), 338 g/L oxadiazon (507.00 g a.i ha^−1^), 330 g/L pendimethalin (144.00 g a.i ha^−1^), and 480 g/L trifluralin (720.00 g a.i ha^−1^). Through Petri dish screening, a spraying treatment was carried out before seed germination. By measuring the root length and shoot length, imazethapyr (100.50 g a.i ha^−1^) and oxadiazon (507.00 g a.i ha^−1^) were screened out. They were applied to potted plants and sprayed after sowing. The results showed that imazethapyr (100.50 g a.i. ha^−1^) and oxadiazon (507.00 g a.i. ha^−1^) had no inhibitory effect on the growth and development of the mung bean. Subsequently, experiments were conducted with imazethapyr (100.50 g a.i ha^−1^) and oxadiazon (507.00 g a.i ha^−1^) applied in the field. Compared to the control, under imazethapyr (100.50 g a.i ha^−1^) and oxadiazon (507.00 g a.i ha^−1^), the agronomic traits, photosynthetic pigment content, yield, and yield components were not inhibited; the activities of superoxide dismutase, peroxidase, and catalase were increased; and gas exchange and chlorophyll fluorescence were not inhibited. In addition, soil urease activity decreased and soil invertase and alkaline phosphatase activity increased after 60 d of treatment. In summary, imazethapyr and oxadiazon can effectively control weeds and increase mung bean yield. The purpose of this study is to screen out safe and efficient pre-emergence herbicides suitable for the Shanxi mung bean, which is of great significance due to its large-scale planting industrialization and the development of advantageous industries.

## 1. Introduction

Mung beans (*Vigna radiata* (Linn) Wilczek.), one of Asia’s most important leguminous crops, have been cultivated in China for more than two thousand years. Mung beans are highly valued for their nutritional content, containing 2–3 times the protein (20–25%) of cereals; a wide range of essential amino acids [1,2,3,4,5,6]; fiber; vitamins A, B, C, and E; minerals, such as calcium, iron, and zinc; and functional factors, such as polyphenols, polysaccharides, and peptides [7]. Alongside improvements in living standards, mung beans are becoming increasingly popular because of their dual use in medicine and food [8]. Recent agricultural supply-side reforms in China have led to increases in the planting area of mung beans [9].

Weeds are a major factor contributing to yield losses in crops, often causing higher economic losses than pests such as insects and fungi. Crops exhibit different competitive abilities for weeds, with the mung bean being a weak competitor, leading to reductions in mung bean yields of up to 87% [10]. During its early growth stage, mung beans grow slowly, making them highly susceptible to competition from weeds, ultimately affecting the yield [11]. The rational use of herbicides during production can improve production efficiency and crop yield [12,13]. Particularly, stem and leaf herbicides, such as imazethapyr, quizalofop-p-ethyl, and fomesafen, have demonstrated good weed control effects in mung bean fields [14]. However, as mung beans are less competitive with weeds in the early stage, making use of pre-emergence herbicides can control weeds earlier on, reducing their effect on the growth of mung beans. Particularly, pendimethalin, prometryn, S-metolachlor, thifensulfuron-methyl, and clomazone are pre-emergence herbicides that have exhibited strong weeding effects [15,16,17]. Kousta et al. [18] showed that flumioxazin had the best weeding effect among the four legumes, followed by benfluralin and the mixture of terbuthylazine plus pendimethalin. Vidal et al. [19] found that 11 pre-emergence herbicides, including acetolactate synthase (ALS), photosystem II (PSII), protoporphyrinogen oxidase (PPO), and very long-chain fatty acid (VLCFA), may have greater benefits in residual weed control than potential concerns for soybean development, nodule formation, and symbiotic nitrogen fixation. Effective herbicides that are safe for mung beans could control broadleaf weeds in mung bean fields, reduce mung bean production costs, and improve production. However, there is no pre-emergence herbicide registration for mung beans on the China Pesticide Information Network. There are only two stem-leaf herbicides (quizalofop-p-ethyl and sulcotrione-oxaloacetate). Consequently, farmers mainly use the same herbicides as for soybeans.

Therefore, this study aims to identify pre-emergence herbicides that are safe and effective for controlling weeds in mung bean fields in Shanxi Province. Specifically, it seeks to evaluate the impact of the weed community and environmental conditions on the weed-control effect and safety of pre-emergence herbicides on mung beans. The results of this study are of great significance for the large-scale cultivation of mung beans and the development of advantageous industries.

## 2. Materials and Methods

### 2.1. Experimental Material

#### 2.1.1. Test Plant Materials

Jin mung bean 9 is a drought-tolerant variety and the primary high-yield, high-quality mung bean variety in production. This was provided by the Institute of Alpine Crops, Shanxi Agricultural University (Jinzhong, China).

#### 2.1.2. Herbicides Tested

Six pre-emergence herbicides registered in soybean fields were selected for pot and field experiments at the recommended doses: 480 g/L alachlor (1935.00 g a.i ha^−1^), 720 g/L metolachlor (1620.00 g a.i ha^−1^), 100 g/L imazethapyr (100.50 g a.i ha^−1^), 338 g/L oxadiazon (507.00 g a.i ha^−1^), 330 g/L pendimethalin (144.00 g a.i ha^−1^), and 480 g/L trifluralin (720.00 g a.i ha^−1^). (The herbicide was selected from the soybean field pre-emergence herbicide registered by China Pesticide Information Network, and the concentration was the recommended dose). The mechanisms of the herbicides are provided in Table 1.

### 2.2. Test Method

#### 2.2.1. Mung Bean Germination Test

The germination test was carried out in an artificial climate chamber (BIC-300) with a constant temperature of 24 °C and humidity of 40%. Double-layer filter paper was placed in Petri dishes with a diameter of 90 mm, and ten uniform and plump seeds were added. After adding 5 mL water, the seeds were sprayed with the six herbicides, with concentrations prepared at 1/1000 of the recommended dose. A 3WP-2000 walking spray tower (developed by Nanjing Institute of Agricultural Mechanization, Ministry of Agriculture and Rural Affairs, Nanjing, China) was used for uniform spraying. Each treatment was repeated three times using a completely randomized design.

#### 2.2.2. Mung Bean Pot Experiment

The mung bean pot experiment was conducted at the Laboratory of Crop Chemical Regulation of Shanxi Agricultural University. A completely randomized design was used. Each treatment was repeated three times. Mung bean seeds were sown in a nutrient bowl (10 × 10 × 15 cm). The soil was a seedling substrate. After sowing, a 3WP-2000 walking spray tower was used to spray the agent. The plants were then placed in a greenhouse and the bottom irrigation method was used for regular replenishment. The morphological indices were measured 7, 14, and 21 d after treatment.

#### 2.2.3. Field Test

**Figure 1:**
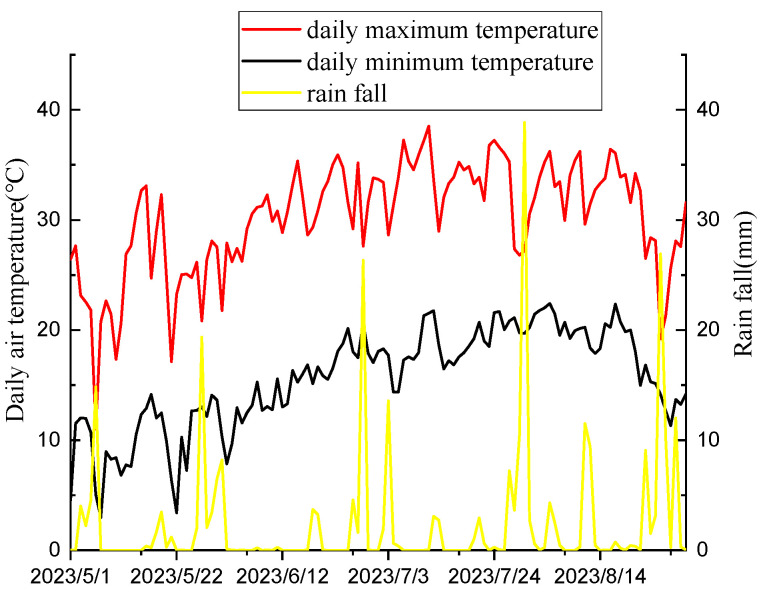
Daily maximum temperature, minimum temperature, and rainfall of the experimental site in 2023.

The field experiment was conducted at the Shenfeng Base of Shanxi Agricultural University (37°42 N, 112°55 E) from May to August 2023. It has a typical temperate continental monsoon climate with an annual average temperature of 9.9 °C. The soil texture is calcareous with PH 7.9. The physical and chemical properties of the soil are organic matter 25.58 g/kg, alkali-hydrolyzable nitrogen 47.94 mg/kg, available phosphorus 23.48 mg/kg, available potassium 129.58 mg/kg, and total zinc 94.89 mg/kg. The previous crop grown in the experimental field was millet. The monthly average maximum and minimum temperatures and monthly precipitation of the test ground in 2023 are shown in Figure 1. Mung beans were sown using a roller hand-push dibbler with a row spacing of 40 cm and plant spacing of 15 cm. The selected recommended doses of imazethapyr (100.50 g a.i ha^−1^) and oxadiazon (507.00 g a.i ha^−1^) were applied to the field and sprayed 2–3 d after sowing. Weed infested (CK) and artificial weeding (CK1) were used as controls. A randomized complete block design was used, and the plot area was 16 m^2^. We repeated the process for each cell three times.

### 2.3. Determination Indexes and Methods

#### 2.3.1. Determination of Mung Bean Germination

The radicle and germ lengths of the seeds were measured using a ruler 7 d after treatment.

#### 2.3.2. Determination of Agronomic Traits of Mung Beans

Plant height, leaf length, leaf width, and root length were measured using a ruler, stem diameter was measured using a Vernier caliper, and aboveground dry weight and root dry weight were measured using a precision balance. The length and width of the two leaves were measured and the leaf area was calculated using the following formula [20]:

Leaf area (cm^2^) = leaf length (cm) × leaf width (cm) × 0.75. (1)

#### 2.3.3. Antioxidant Enzyme Activity of Mung Bean Root

To determine the root antioxidant enzyme activity, 0.1 g of root was ground in a grinding machine and 2 mL of pH 7.8 phosphate buffer was added, mixed with a vortex instrument, centrifuged at 4 °C for 15 min in a low-temperature high-speed centrifuge at 12,000 r/pm, and placed in a refrigerator at 4 °C. Superoxide dismutase (SOD) activity was determined using the NBT photochemical reduction method [21], the activity of peroxidase (POD) activity was determined using the guaiacol oxidation method [21], and catalase (CAT) activity was determined using the hydrogen peroxide method [21].

#### 2.3.4. Determination of Soil Enzyme Activity

Samples were taken at 3, 7, 14, 21, 40, and 60 d after administration. Five soil samples (0–20 cm) were collected from each plot using the diagonal five-point sampling method. Soil samples from the five sampling points were mixed, placed in sterile sealed bags, and brought to the laboratory as soon as possible. Soil samples were ground immediately after removing impurities such as stones and plant roots.

The soil urease activity was determined using the phenol-sodium hypochlorite colorimetric method. The activity was expressed as the number of micrograms of NH4-N produced per gram of dry soil after incubation at 37 °C for 24 h, and the wavelength was 578 nm [22]. The soil sucrase activity was determined using the 3,5-dinitrosalicylic acid colorimetric method. The activity was expressed as the number of milligrams of glucose produced per gram of dry soil after incubation at 37 °C for 24 h, and the wavelength was 540 nm [22]. Soil alkaline phosphatase activity was determined using the disodium phenyl phosphate colorimetric method. The activity was expressed as the amount of phenol released per gram of dry soil after incubation at 37 °C for 24 h, and the wavelength was 660 nm [22].

#### 2.3.5. Photosynthetic Pigment Content, Gas Exchange, and Chlorophyll Fluorescence Parameters of Mung Beans at Seed-Filling Stage

The photosynthetic pigment content was determined using the ethanol extraction method. A 0.05 g sample of the third leaf of mung bean was placed in a centrifuge tube, and 5 mL 96% ethanol was added and soaked for 24 h in the dark. When the leaves were white, the absorbance values at 665, 649, and 470 nm were measured using a microplate reader. The photosynthetic pigment content was calculated using the following formulas:

Chlorophyll a = 13.95 × A_665_ − 6.88 × A_649._ (2)

Chlorophyll b = 24.96 × A_649_ − 7.32 × A_665._ (3)

Carotenoids = (1000 × A_470_ − 2.05 × chlorophyll a − 114.8 × chlorophyll b)/245. (4)

Gas exchange parameters were measured using a photosynthetic instrument CI340 (Aidi Ecological Science Instruments Co., Ltd., WA, USA). On sunny cloudless days at 9:00–11:00, the net photosynthetic rate, stomatal conductance, transpiration rate, and intercellular CO_2_ concentration of mung bean leaves were measured.

Chlorophyll fluorescence parameters were measured using a portable pulse-modulated chlorophyll fluorometer (PAM-2500; WALZ, Effeltrich, Germany). The actual photosynthetic efficiency, photochemical quenching coefficient, non-photochemical quenching coefficient, and maximum photochemical efficiency of Photosystem II were measured after the leaves were subjected to sufficient dark adaptation (more than 30 min).

#### 2.3.6. Yield and Yield Components at Harvest Time

During the harvest period, a square area of 1 × 1 m for yield measurement was selected in each plot and the average value of each treatment was converted into hectare yield. The number of branches, number of pods per plant, number of effective pods per plant, and 100-seed weight were measured using three labeled plants as samples.

#### 2.3.7. Field Weed Control Effect

The field weed control effect was investigated at 10, 20, 30, and 40 d after treatment. The five-point sampling method was used to investigate the number and fresh weight of weeds in each plot of 0.25 m^2^ (0.5 × 0.5) and the plant control effect and dry weight control effect were calculated as follows:

Plant control effect (%) = (number of weeds in weed infested control area-number of weeds in treatment area)/number of weeds in weed infested control area × 100 (%).(5)

Dry weight control effect (%) = (weed dry weight in weed infested control area − weed dry weight in treatment area)/weed dry weight in weed infested control area × 100 (%).(6)

### 2.4. Data Analysis

All experiments were completely randomized, and each treatment was repeated three times. Duncan’s new complex range analysis of variance and multiple comparisons were performed using SPSS (25.0, IBM SPSS Inc., Chicago, IL, USA) software (*p* < 0.05 was considered statistically significant). Graph Pad Prism (8.0.2, Graph Pad Software, San Diego, CA, USA) was used for graphical analysis.

## 3. Results

### 3.1. Effect of Pre-Emergence Herbicides on Mung Bean Germination

**Figure 2:**
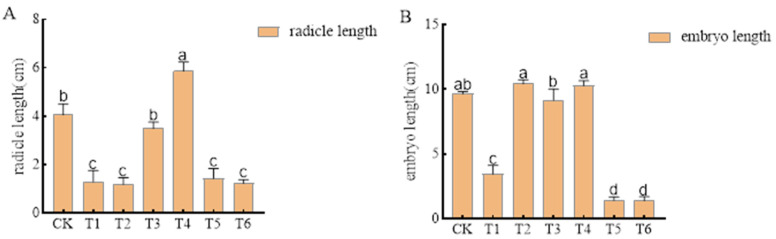
Effects of pre-emergence herbicides on (**A**) radicle length and (**B**) germ length of mung bean seeds.

Pre-emergence herbicides alachlor (1935.00 g a.i ha^−1^), metolachlor (1620.00 g a.i ha^−1^), imazethapyr (100.50 g a.i ha^−1^), oxadiazon (507.00 g a.i ha^−1^), pendimethalin (144.00 g a.i ha^−1^), and trifluralin (720.00 g a.i ha^−1^) had no effect on mung bean seed germination, achieving a germination rate of 100%. However, most had significant effects on the radicle and germ length (Figure 2). The radicle lengths under alachlor (1935.00 g a.i ha^−1^), metolachlor (1620.00 g a.i ha^−1^), pendimethalin (144.00 g a.i ha^−1^), and trifluralin (720.00 g a.i ha^−1^) were 68.03%, 70.49%, 64.75%, and 69.67% lower than that of unsprayed treatment control, respectively. The germ lengths under alachlor (1935.00 g a.i ha^−1^), pendimethalin (144.00 g a.i ha^−1^), and trifluralin (720.00 g a.i ha^−1^) were 64.14%, 85.17%, and 85.52% lower than that of unsprayed treatment control, respectively. In contrast, imazethapyr (100.50 g a.i ha^−1^) and oxadiazon (507.00 g a.i ha^−1^) had no significant effects on radicle and germ length.

### 3.2. Effects of Pre-Emergence Herbicides on Agronomic Traits of Mung Bean Potted Plants

**Figure 3:**
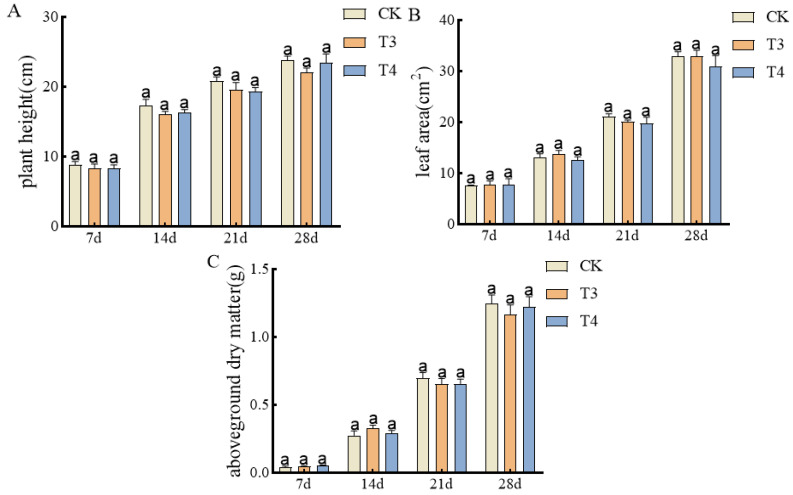
Effects of pre-emergence herbicides on agronomic traits of mung bean pots: (**A**) plant height, (**B**) leaf area, and (**C**) aboveground dry weight.

Safe pre-emergence herbicides selected from the Petri dishes were applied to the pots. As shown in Figure 3A, there was no significant difference in plant height between imazethapyr (100.50 g a.i ha^−1^) and oxadiazon (507.00 g a.i ha^−1^) from 7 to 28 d of treatment. Also, there was no significant difference in leaf area between unsprayed treatment control and imazethapyr (100.50 g a.i ha^−1^) and oxadiazon (507.00 g a.i ha^−1^) (Figure 3B). There was no significant difference in the above-ground dry weight (Figure 3C).

### 3.3. Effects of Pre-Emergence Herbicides on Agronomic Traits of Mung Beans in the Field

**Figure 4:**
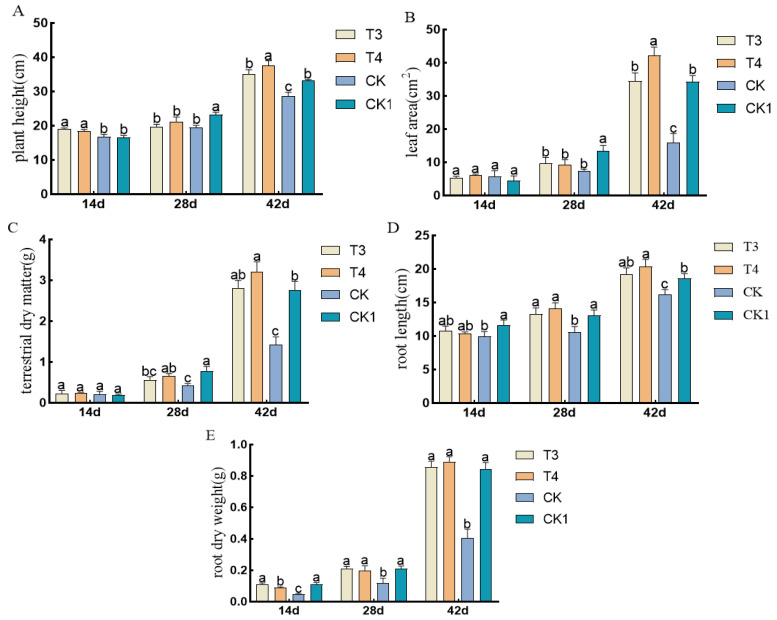
Effects of pre-emergence herbicides on agronomic traits of mung beans: (**A**) plant height, (**B**) leaf area, (**C**) aboveground fresh weight, (**D**) root length, and (**E**) root dry weight.

The pre-emergence herbicides screened in the laboratory were applied in the field. Under imazethapyr (100.50 g a.i ha^−1^) and oxadiazon (507.00 g a.i ha^−1^), the plant height, leaf area, aboveground dry weight, root length, and root dry weight of mung beans were not inhibited compared to those of artificial weeding (CK1) (Figure 4).

### 3.4. Effects of Pre-Emergence Herbicides on Antioxidant Enzyme Activities in Mung Bean Roots

**Figure 5:**
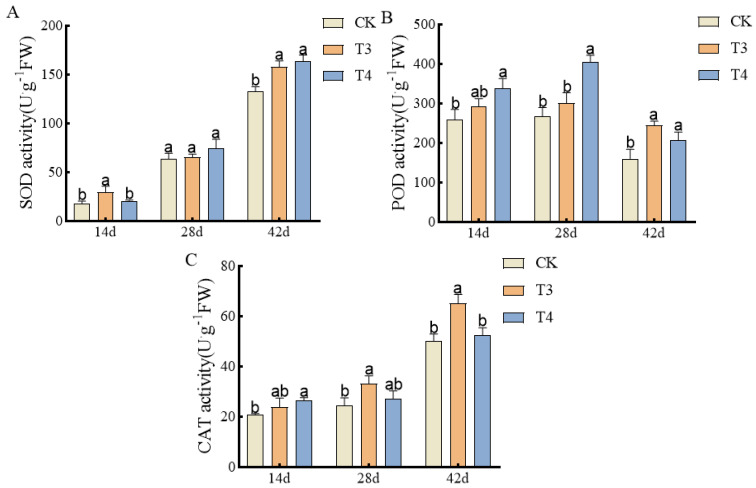
Effects of pre-emergence herbicides on antioxidant enzyme activities in roots of mung bean plants: (**A**) SOD activity, (**B**) POD activity, and (**C**) CAT activity.

With time after drug administration, the activities of SOD and CAT showed an overall upward trend. The activity of POD showed a trend of first increasing and then decreasing (Figure 5). At 14 d after treatment, the SOD activity under imazethapyr (100.50 g a.i ha^−1^) was significantly increased by 40.11% compared with weed infested (CK). At 28 d, there was no significant difference under imazethapyr (100.50 g a.i ha^−1^), oxadiazon (507.00 g a.i ha^−1^), and weed infested (CK). At 42 d, the SOD activity under imazethapyr (100.50 g a.i ha^−1^) and oxadiazon (507.00 g a.i ha^−1^) was significantly increased by 15.89% and 18.74% compared with weed infested (CK) (Figure 5A). At 14 d, the POD activity under oxadiazon (507.00 g a.i ha^−1^) was significantly higher than that of weed infested (CK) by 23.37%. At 28 d, the POD activity under imazethapyr (100.50 g a.i ha^−1^) was significantly higher than that of weed infested (CK) by 33.89%. At 42 d, imazethapyr (100.50 g a.i ha^−1^) and oxadiazon (507.00 g a.i ha^−1^) were significantly higher than weed infested (CK) by 34.56% and 22.53% (Figure 5B). At 14 d, the CAT activity under oxadiazon (507.00 g a.i ha^−1^) was significantly increased by 21.46% compared with weed infested (CK). At 28 d, the CAT activity under imazethapyr (100.50 g a.i ha^−1^) was significantly increased by 26.51% compared with weed infested (CK). At 42 d after treatment, imazethapyr (100.50 g a.i ha^−1^) was significantly increased by 23.31% compared with weed infested (CK) (Figure 5C).

### 3.5. Soil Enzyme Activity

**Figure 6:**
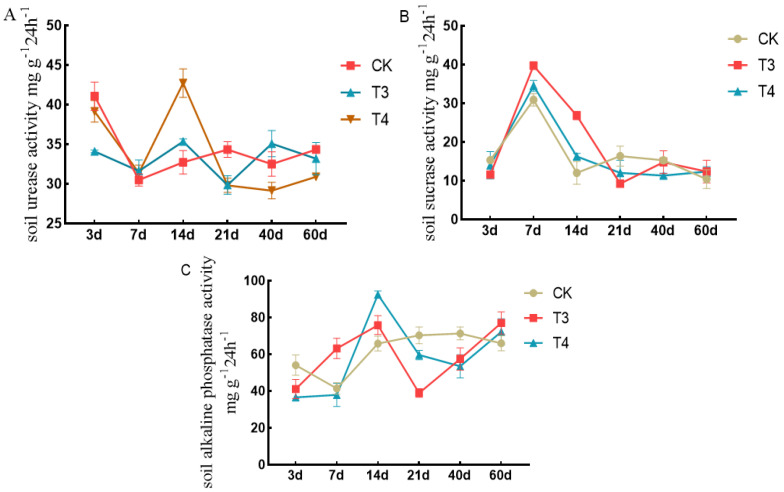
Effects of pre-emergence herbicides on soil enzyme activity: (**A**) soil urease, (**B**) soil invertase, and (**C**) soil alkaline phosphatase.

The dynamic responses of soil urease to the different herbicides are shown in Figure 6A. The results showed that the enzyme activity in each treatment first decreased, then increased, and then decreased from 3 to 60 d. The urease activity of each treatment group decreased from 3 to 7 d, increased from 7 to 14 d, and showed a small upward trend of 21–60 d. At 14 d, the urease activities under imazethapyr (100.50 g a.i ha^−1^) and oxadiazon (507.00 g a.i ha^−1^) increased by 7.47% and 26.01%, respectively, compared with weed infested (CK).

The dynamic responses of soil sucrase to the different herbicides are shown in Figure 6B. The results showed that the enzyme activity of each treatment first increased and then decreased from 3 to 60 d. The enzyme activity of each treatment increased from 3 to 7 d, decreased from 7 to 14 d, and was stable from 14 to 60 d. The enzyme activity peaked at 7 d. The sucrase activity under imazethapyr (100.50 g a.i ha^−1^) and oxadiazon (507.00 g a.i ha^−1^) increased by 22.25% and 10.52%, respectively, compared with weed infested (CK).

The dynamic responses of soil alkaline phosphatase to different herbicides are shown in Figure 6C. At 3 d, the alkaline phosphatase activity under imazethapyr (100.50 g a.i ha^−1^) and oxadiazon (507.00 g a.i ha^−1^) decreased by 24.03% and 32.41%, respectively, compared with weed infested (CK). At 60 d, the enzyme activities under imazethapyr (100.50 g a.i ha^−1^) and oxadiazon (507.00 g a.i ha^−1^) increased by 14.31% and 8.58% compared with weed infested (CK).

### 3.6. Effects of Pre-Emergence Herbicides on Photosynthetic Pigment Content, Gas Exchange, and Chlorophyll Fluorescence Parameters of Mung Beans at the Seed-Filling Stage

**Figure 7:**
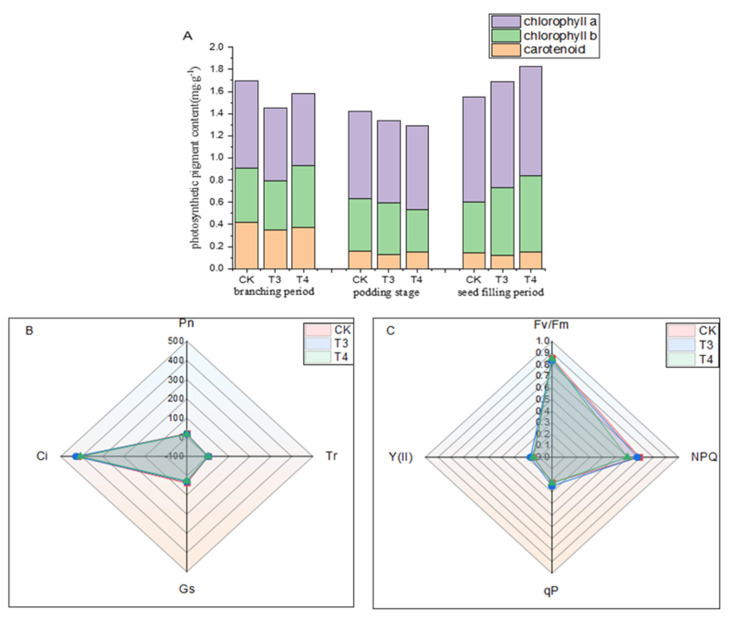
Effects of pre-emergence herbicides on (**A**) photosynthetic pigment content, (**B**) gas exchange, and (**C**) chlorophyll fluorescence parameters of mung beans at filling stage. Pn: net photosynthetic rate; Ci: intercellular CO_2_ concentration; Gs: stomatal conductance; Tr: transpiration rate; Y (II): actual photosynthetic efficiency; qP: photochemical quenching coefficient; NPQ: non-photochemical quenching coefficient; Fv/Fm: maximum photochemical efficiency.

Photosynthesis is the basis of mung bean yield, and the seed-filling stage is the key period for seed growth and filling. Most of the organic matter synthesized by photosynthesis is transported to the seed and converted into starch and storage proteins, which are the main components of seed yield. Photosynthesis during the seed-filling stage plays a decisive role in the yield. Figure 7A shows that compared with weed infested (CK), imazethapyr (100.50 g a.i ha^−1^) and oxadiazon (507.00 g a.i ha^−1^) increased chlorophyll a by 0.84% and 4.32% and chlorophyll b by 26.18% and 34.49%. There were no significant differences in the net photosynthetic rate, intercellular CO_2_ concentration, stomatal conductance, or transpiration rate (Figure 7B). There were no significant differences in the maximum photochemical efficiency, actual photosynthetic efficiency, photochemical quenching coefficient, and non-photochemical quenching coefficient (Figure 7C).

### 3.7. Effects of Pre-Emergence Herbicides on Yield and Yield Components of Mung Bean

As shown in Table 2, there was no significant difference in the number of branches, pods per branch, effective pods per branch, 100-seed weight, and yield between imazethapyr (100.50 g a.i ha^−1^), oxadiazon (507.00 g a.i ha^−1^), and artificial weeding (CK1), and the two pre-emergence herbicides did not affect the yield and yield components of mung beans.

### 3.8. Weed Control Effect

**Figure 8:**
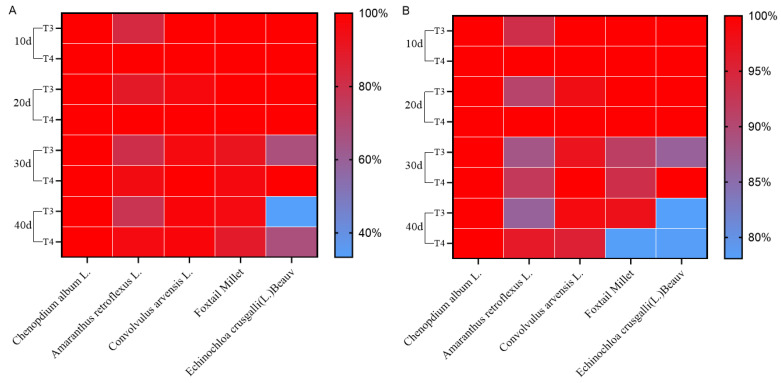
Effects of pre-emergence herbicides on weed (**A**), plant control, and (**B**) dry weight control.

As shown in Figure 8, imazethapyr (100.50 g a.i ha^−1^) and oxadiazon (507.00 g a.i ha^−1^) had a positive effect on the overall weed control. Field weed species are mainly broad-leaved species, such as *Chenopodium*, *Amaranthus retroflexus*, *Convolvulus*, Gramineae (millet), and *Echinochloa crusgalli*. The control effects of imazethapyr (100.50 g a.i ha^−1^) and oxadiazon (507.00 g a.i ha^−1^) on the plant and dry weight of *Chenopodium album* were 100% at 10, 20, 30, and 40 d after treatment. The control effect on *A. retroflexus* exceeded 80% after 10–30 d under imazethapyr (100.50 g a.i ha^−1^) treatment and 78.26% after 40 d. The dry weight control effect on *A. retroflexus* was more than 80% after 10–40 d of treatment. The dry weight control effects on *Convolvulus japonicus* and *Setaria italica* exceeded 90% after 40 d of treatment. The dry weight control effect on *Echinochloa crus-galli* was 66.67% and 86.63% after 30 d of treatment. The control effect of oxadiazon (507.00 g a.i ha^−1^) on *A. retroflexus* was still greater than 90% after 40 d of treatment and that on *Convolvulus arvensis* was 100% after 30 d of treatment. The control effect on gramineous millet (89.01%) plants was greater than that on barnyard grass (66.67%) after 40 d of treatment.

## 4. Discussion

Weeds are a major threat to global food production [23], competing with crops for light, water, and nutrition, which restricts the high and stable yield of crops. Despite an annual investment of CNY 235 billion for prevention and control, weeds still cause direct economic losses of nearly CNY 100 billion for grain in China [24]. Despite the effectiveness of chemical weeding, no pre-emergence herbicides have been registered for mung bean fields in the China Pesticide Information Network. Victor et al. [25] found that ALS-inhibiting herbicides still had control value on other weeds in the soil seed bank, except for the resistant populations. This study showed that imazethapyr and oxadiazon exhibited good weed-control effects, consistent with the results of previous studies which showed that imazethapyr could replace manual weeding before seeding [26].

However, the use of herbicides can result in an inappropriate living environment for crops. In response to stress, plants can activate their antioxidant systems, such as SOD, CAT, and POD [27,28,29]. Wang et al. [30] showed that a combination of herbicides and safeners significantly increased the activities of SOD and POD, which is consistent with the results of the present study. In this study, the activities of SOD, POD, and CAT were significantly increased after spraying with imazethapyr and oxadiazon compared to the control, indicating that mung beans can synergistically protect themselves under herbicide stress.

The photosynthetic pigment content plays an important role in gas exchange and chlorophyll fluorescence. Zili et al. [31] showed that short-term stress after herbicide application would increase chlorophyll content and photosynthetic capacity. Another study found that herbicides did not affect the chlorophyll fluorescence of citrus seedlings; however, the transpiration rate decreased [32]. In the present study, the chlorophyll content was inhibited compared with the control at the early stage of imazethapyr and oxadiazon application, the photosynthetic pigment content was not inhibited at the seed-filling stage, and the gas exchange and chlorophyll fluorescence parameters at the seed-filling stage were not significantly different from those of the control.

Soil enzymatic activity is an important indicator of the ecological environment. At the recommended dose, the soil urease activity of imazethapyr and oxadiazon decreased and the soil invertase and alkaline phosphatase activities increased. Nicoleta et al. [33] showed that soil enzyme activity was inhibited after treatment with the recommended dose of oxyfluorfen. The recovery of phosphatase, urease, and protease activity required a minimum of 21 d, while dehydrogenase activity was restored after 14 d. The application of herbicides activated the soil enzyme activity at the beginning of the incubation period. This may be because herbicides are equivalent to the carbon or nitrogen matrices that can be used by soil microorganisms [34,35]. However, our research mainly focused on the changes in soil enzyme activities when using pre-emergence herbicides. It is necessary to further study how these treatments affect other influencing factors, such as soil microbial community structure, to fully understand the changes in the soil environment under pre-emergence herbicide stress.

Nikola et al. [36] discovered that mesotrione did not result in a decrease in soybean yield, but it had the potential to cause early damage to crops. In this study, imazethapyr and oxadiazon were screened out of six herbicides by root length and bud length using Petri dishes. The recommended dose was applied to pots and found to have no inhibitory effect on the growth of mung beans and then used in the field. Under field application, both herbicides showed effective weed control with minimal impacts on the agronomic traits and yield of mung beans. Therefore, we can preliminarily suggest these herbicides for use in mung bean fields. However, owing to a single point of the test in a single year, multiple verifications are required. Follow-up studies will provide these verifications and contribute to the large-scale planting of mung bean industrialization and the development of characteristic and advantageous industries.

## 5. Conclusions

In the indoor experiment, six kinds of pre-emergence herbicides were sprayed on the seeds, and imazethapyr (100.50 g a.i ha^−1^) and oxadiazon (507.00 g a.i ha^−1^) were screened by seed germination radicle length and germ length, which did not affect the growth of mung bean plants after being applied to pots. Then, it was applied to the field and was found to have a good effect on weed control in the mung bean field, improving the activity of antioxidant enzymes and reducing the damage to the cell membrane system. It did not affect the growth, leaf green content, gas exchange, and chlorophyll fluorescence of the mung beans, thereby increasing its yield. The increase in soil sucrase and alkaline phosphatase contributes to the restoration of soil biochemical stability. Therefore, the application of imazethapyr (100.50 g a.i ha^−1^) and oxadiazon (507.00 g a.i ha^−1^) in the field is beneficial to increase the yield of mung bean. This study provides a certain basis and scientific theoretical research for screening out safe and effective pre-emergence herbicides for weeds in mung bean fields in Shanxi Province.

## Figures and Tables

**Table 1:** Herbicides tested and their mechanism of action.

Treatment	Common Name	Trade Name	Manufacturer	Molecular Target	Application Rate(g a.i ha^−1^)
T1	43% alachlor	Lasso	Jiangsu Nantong Jiangshan Pesticide Chemical Co., Ltd. (Jiangsu, China).	Cell division	1935.00
T2	720 g/L metolachlor	Dual	Shandong Sannong Biotechnology Co., Ltd. (Shandong, China).		1620.00
T3	10% imazethapyr	Pursuit	Jiangsu Ruibang Agrochemical Co., Ltd. (Jiangsu, China).	Acetolactate synthetase	100.50
T4	26% oxadiazon	Ronstar	Anhui Kelihua Chemical Co., Ltd. (Anhui, China).	Protoporphyrinogen oxidase	507.00
T5	330 g/L pendimethalin	Stomp	Shandong Sannong Biotechnology Co., Ltd. (Shandong, China).	Microtubule assembly	144.00
T6	480 g/L trifluralin	Flutrix 48EC	Qiaochang Modern Agriculture Co., Ltd. (Shandong, China).		720.00

**Table 2:** Effects of pre-emergence herbicides on yield components and yield of mung beans in the field.

Variety	Treatment	Number of Branching	Number of Pods per Branch	Number of Effective Pods per Branch	100-Seed Weight (g)	Yield (kg/hm^2^)
Jin mung bean 9	T3	5.33 ± 0.33 a	26.64 ± 0.28 a	26.19 ± 0.24 a	7.20 ± 0.21 a	1695.52 ± 42.34 a
	T4	5.67 ± 0.33 a	25.52 ± 0.48 a	24.96 ± 0.82 a	7.00 ± 0.17 a	1673.17 ± 41.09 a
	CK	2.33 ± 0.33 b	16.21 ± 0.29 b	15.76 ± 0.32 b	7.06 ± 0.19 a	555.75 ± 15.54 b
	CK1	5.83 ± 0.44 a	24.43 ± 1.45 a	23.68 ± 1.21 a	7.86 ± 0.60 a	1733.43 ± 40.06 a

Note: Experimental data were presented as mean ± standard deviation (SD) (n = 3). Different lowercase letters after the numbers in the same column indicate significant differences at *p* < 0.05 by the LSD method. T3, imazethapyr (100.50 g a.i ha^−1^); T4, oxadiazon (507.00 g a.i ha^−1^); CK, weed infested; CK1, artificial weeding.

## Data Availability

All data generated or analyzed in this study are included in this published article. For further information, please contact the corresponding author.
